# Analgesic efficacy of a bilateral erector spinae plane block versus a fentanyl constant rate infusion in dogs undergoing hemilaminectomy: a retrospective cohort study

**DOI:** 10.1186/s12917-022-03523-x

**Published:** 2022-12-05

**Authors:** Inga Viilmann, Maja Drozdzynska, Enzo Vettorato

**Affiliations:** 1 Dick White Referrals, part of Linnaeus Veterinary Limited, Station Farm, London Road, Six Mile Bottom, Cambridgeshire, CB8 0UH UK; 2 Small Animal Specialist Hospital, Level 1, 1 Richardson Place, North Ryde, NSW 2113 Australia; 3 Department of Comparative, Diagnostic, and Population Medicine, College of Veterinary Medicine, University of Florida, FL Gainesville, USA

**Keywords:** Dog, Erector spinae plane block, Hemilaminectomy, Regional anaesthesia, Analgesia

## Abstract

**Background:**

Erector spinae plane block (ESPB) is an ultrasound-guided interfascial plane block used to provide analgesia in dogs undergoing hemilaminectomy. The aim of this study is to compare the analgesic efficacy of a bilateral ESPB with a fentanyl constant rate infusion (CRI) in dogs undergoing hemilaminectomy.

**Methods:**

This is a retrospective cohort study. Anaesthetic records of client-owned dogs undergoing hemilaminectomy (June 2019–August 2020), and in which a bilateral ESPB was performed (group ESPB), were retrieved and compared to a cohort of 39 dogs that underwent hemilaminectomy (September 2014 – June 2017) and in which a fentanyl CRI (2 μg/kg bolus followed by 5 μg/kg/hour) was used as a primary intraoperative analgesia (group CRI). The prevalence of dogs in which intraoperative rescue fentanyl boluses were administered, the total dose of rescue fentanyl boluses administered, the postoperative methadone requirement and anaesthetic complications during the first 24 postoperative hours were evaluated. Univariate statistical analysis was used.

**Results:**

Group ESPB comprised of 93 dogs. The bilateral ESPB was performed using a median (range) levobupivacaine volume of 1 (0.5–1.7) mL/kg *per* side, at a concentration of 0.125% (0.12–0.25). At least one rescue fentanyl bolus was administered in 54.8% and in 56.4% of dogs in group ESPB and CRI, respectively (*p* > 0.99). The number of rescue fentanyl boluses was higher in group CRI (*p* = 0.006), especially during lumbar hemilaminectomy. Rescue fentanyl boluses were more frequently administered from skin incision to end of vertebral lamina drilling in group CRI (*p* = 0.001), and from end of vertebral lamina drilling to end of surgery in group ESPB (*p* = 0.0002). During the first 6 (*p* = 0.0035) and 6–12 (*p* = 0.0005) postoperative hours, the number of dogs that required at least one dose of methadone was higher in group CRI. In group ESPB, dogs were more likely to become hypothermic (*p* = 0.04). One dog, not included in the study, developed sinus arrest after performing a caudal thoracic ESPB.

**Conclusions:**

Under the conditions of this study, a bilateral ESPB was associated with a lower number of rescue fentanyl boluses administered in dogs undergoing hemilaminectomy, especially between skin incision to end of vertebral lamina drilling. Despite ESPB being associated with a reduced opioid consumption during the first 12 hours postoperatively, differences in the postoperative management precluded any firm conclusion regarding its postoperative effect.

## Background

Erector spinae plane block (ESPB) is an ultrasound-guided interfascial plane block in which a local anaesthetic is deposited to the thoracolumbar fascia at the level of transverse processes of the vertebrae. Despite its mechanism of action is not being fully understood, it is most likely related to a direct effect of the local anaesthetic to neural structures in the fascial plane deep to the erector spinae muscles, and adjacent tissue compartments [1]. ESPB provided intraoperative analgesia for thoracotomies, laparotomies and spinal surgeries in humans [2].

Thoracic and lumbar ESPB has been described in canine, equine and porcine cadaveric studies and has consistently stained the dorsal branches of the spinal nerve in canine cadaveric studies [3–8]. In dogs and cats undergoing hemilaminectomy, ESPB provided analgesia [9–12] and reduced the intraoperative pharmacological interventions to treat cardiovascular complications [13].

This retrospective cohort study compares the analgesic efficacy of a bilateral ESPB and a fentanyl constant rate infusion (CRI) in dogs undergoing hemilaminectomy. We hypothesised that the total number of intraoperative rescue fentanyl administrations (main outcome), and the number of animals requiring opioid during the first 6 postoperative hours (secondary outcome), would be lower when the ESPB was performed.

## Methods

Due to the observational nature of this retrospective cohort study, ethical approval was not pursued. Signed informed owner consent to use clinical information for retrospective studies was granted at the time of the animal’s admission. All methods were carried out in accordance with relevant local guidelines and regulations.

Dogs that underwent thoracic or lumbar hemilaminectomy for disc(s) extrusion or protrusion at Dick White Referrals (UK), between June 2019 and August 2020, and in which a preoperative bilateral ESPB was performed (group ESPB), were retrieved. Medical records were excluded if: the volume of the local anaesthetic used on each side for the ESPB was < 0.5 mL/kg; if fentanyl (Fentadon; Dechra Pharmaceuticals, UK) was not the only rescue analgesic drug used intraoperatively; if additional surgeries other than hemilaminectomy were performed; if the dog was scored 5 (paraplegia without deep pain) according to the modified Frankel score (MFS) [14]; if the dog experienced any possible adverse reaction to the drug used; if medical records were incomplete or data were missing. The control group (group CRI) was composed of 39 dogs that underwent hemilaminectomy between September 2014 and June 2017, and in which a fentanyl CRI (2 μg/kg bolus followed by 5 μg/kg/hour) was administered [15].

The ESPB was performed using a previously described technique by several anaesthetists with different level of experience in ultrasound-guided fascial plane blocks [3]. All dogs were positioned in sternal recumbency; the transverse process of the vertebrae was identified ultrasonographically using a linear probe (5–13 MHz; SonoScape S6V, SonoScape, UK) and a parasagittal approach. A 20–22 gauge Tuohy needle (Perican®; Braun, Germany) of adequate length was directed in-plane to target the transverse process. Levobupivacaine (Chirocaine 0.5%; AbbVie Srl, Italy) was injected while observing the sonographic hydro-dissection of desired fascial plane and the characteristic elevation of the epaxial muscles. The volume and the concentration of levobupivacaine was decided by the anaesthetist in charge of the case.

The information reported in Tables 1, 2 and 3 were collected and logged in a Microsoft Excel spread sheet (Microsoft Corp, v 16.41, USA). The number of rescue IV fentanyl boluses administered by the attending anaesthetist to control a sudden increase in heart rate (HR) and/or mean arterial blood pressure (MAP) possibly related to nociception, in the absence of any previous pharmacological intervention or any other abnormality that would suggest otherwise, and which subsided after the administration of fentanyl, was recorded. The overall intraoperative rescue fentanyl administered (μg/kg/hour) was calculated by adding the amount of rescue fentanyl (μg) injected during the surgery and dividing by the animal’s body weight (kg) and by surgical time (hour). The time of the first nociceptive event was considered either from skin incision to end of drilling of the vertebral lamina (phase 1) or from end of drilling of the vertebral lamina to end of surgery (phase 2). Surgeries were defined: “thoracolumbar” if the herniated disc was caudal to the tenth thoracic vertebra (T10) and cranial to the first lumbar vertebra (L1), “lumbar” if the herniated disc was caudal to L1 but cranial to L6.

**Table 1:** Demographical data, anaesthetic and surgical details of 132 dogs undergoing thoracolumbar (cranial to the 1st lumbar vertebra) or lumbar (caudal to the 1st lumbar vertebra) hemilaminectomy surgery and in which a bilateral erector spinae block (group ESPB) or a fentanyl infusion (group CRI) was used. Data are reported as mean ± standard deviation or median (range). Odd ratio (OR) and 95% Confidence intervals (CI) is reported when appropriate. * indicates reciprocal OR

Group		ESPB (***n*** = 93)	CRI (***n*** = 39)	ESPB vs CRI ***p*** value	OR (95% CI)
Information					
Age (months)		70 ± 34	71 ± 29	0.92	
Sex (n. of dogs)		10 F (10.7%) 33 FS (35.5%) 18 M (19.4%) 32 MN (34.4%)	2 F (5.1%) 11 FS (28.2%) 8 M (20.5%) 18 MN (46.2%)	0.18	
Body weight (kg)		10 (3.1–38.3)	8.6 (3.9–41)	0.29	
MFS status (n. of dogs)		35 MFS-1 or 2 (37.6%) 53 MFS-3 (57%) 5 MFS-4 (5.4%)	31 MFS-1 or 2 (79.5%) 8 MFS-3 (20.5%) 0 MFS-4 (0%)	< 0.0001	5.87 (2.39–13.70) *
Pre-anaesthetic medication	Methadone (n. of dogs)	93 (100%)	39 (100%)	1	
	Methadone dose (mg/kg)	0.2 (0.1–0.3)	0.25		
	Acepromazine (n. of dogs)	1 (1.1%)	0	1	
	Acepromazine (mg/kg)	0.01			
	Dexmedetomidine (n. of dogs)	89 (95.7%)	0	< 0.0001	
	Dexmedetomidine (μg/kg)	1 (0.5–2)			
Time from premedication to start of surgery (min)		85 (35–240)	90 (60–150)	0.18	
Induction agent	Propofol (n. of dogs)	85 (91.4%)	39 (100%)	0.10	
	Propofol (mg/kg)	2.4 (0.9–5.4)	4.2 (2.4–7.8)	< 0.0001	
	Alfaxalone (n. of dogs)	8 (8.6%)	0	0.10	
	Alfaxalone (mg/kg)	0.9 (0.7–1.8)			
Maintenance of anaesthesia (n. of dogs)		90 Isoflurane (96.8%) 3 Sevoflurane (3.2%)	39 Isoflurane (100%) 0 Sevoflurane (0%)	0.55	
MAC multiple IAA		0.94 (0.86–1.1)	1.08 (0.91–1.19)	< 0.0001	
Type of disc herniation (n.)		91 extrusions (97.8%) 2 protrusions (2.2%)	39 extrusions (100%) 0 protrusions (0%)	1	
Lateralisation of herniated disc (n.)		51 left (54.8%) 42 right (45.2%)	23 left (59%) 16 right (41%)	0.70	
Herniated intervertebral disc space (n.)		80 1-disc space (86%) 10 2-disc spaces (10.8%) 3 3-disc spaces (3.2%)	39 1-disc space (100%) 0 2-disc spaces (0%) 0 3-disc spaces (0%)	0.01	
Type of surgery	Thoracolumbar (n. of dogs)	62 (66.7%)	21 (53.8%)	0.17	
	Lumbar (n. of dogs)	31 (33.3%)	18 (46.1%)		
Disc fenestration (n.)		91 (97.8%)	36 (92.3%)	0.15	
Number of discs fenestrated		3 (1–5)	4 (1–6)	< 0.0001	
Surgical time (min)		70 (30–150)	65 (40–110)	0.17	
Anaesthesia time (min)		161 ± 43	140 ± 31	0.01	

*n.* number; vs versus; *F* female; *FS* female spayed; *M* male; *MN* male neutered; *MFS* modified Frankel scale; *MAC* minimum alveolar concentration; *IAA* inhalational anaesthetic agent; *min* minute

**Table 2:** Intraoperative rescue fentanyl bolus administration and postoperative analgesia during the first 24 hours after surgery of 132 dogs undergoing thoracolumbar (cranial to the 1st lumbar vertebra) or lumbar (caudal to the 1st lumbar vertebra) and in which a bilateral erector spinae block (group ESPB) or a fentanyl infusion (group CRI) was used. Fentanyl administration and dosage, time from start of surgery to first rescue fentanyl bolus administration and methadone dosage are reported as median (range). Odd ratio (OR) and 95% Confidence intervals (CI) is reported when appropriate. * indicates reciprocal OR

Group			ESPB (n = 93)	CRI (n = 39)	ESPB vs CRI ***p*** value	OR (95% CI)
Information						
Rescue fentanyl bolus administration (n. of dogs)			51 (54.8%)	22 (56.4%)	> 0.99	
Rescue fentanyl bolus administration during thoracolumbar surgery (n. of dogs)			37 (39.8%)	7 (18%)	0.13	2.40 (0.85–6.93)
Rescue fentanyl bolus administration during lumbar surgery (n. of dogs)			14 (15%)	14 (36%)	0.037	4.25 (1.08–13.56) *
Number of rescue fentanyl boluses administered			1 (1–4)	2 (1–7)	0.006	
Total rescue fentanyl dose (μg/kg/hour)			1.7 (0.4–6)	1.6 (0.75–5.1)	0.59	
Time from start of surgery to first rescue fentanyl bolus administration (min)			25 (5–105)	10 (5–50)	< 0.0001	
Postoperative oral analgesia (n. of dogs)		Gabapentin, NSAID	71 (76.3%)	39 (100%)	0.0008	
		Gabapentin, NSAID, amantadine	1 (1.1%)	0 (0%)	1	
		Gabapentin, NSAID, paracetamol	1 (1.1%)	0 (0%)	1	
		Gabapentin	13 (13.9%)	0 (0%)	0.01	
		Gabapentin, paracetamol	2 (2.2%)	0 (0%)	1	
		Gabapentin, steroid	2 (2.2%)	0 (0%)	1	
		Gabapentin, steroid, paracetamol	3 (3.2%)	0 (0%)	0.55	
Postoperative methadone requirement	First 6 hours	At least one dose administrated (n.)	15 / 86 (17.4%)	17 (43.6%)	0.003	3.66 (1.60–8.71) *
		Dose (mg/kg)	0.2 (0.2–0.5)	0.2 (0.2–0.4)	0.18	
	6–12 hours	At least one dose administrated (n. of dogs)	9 / 86 (10.5%)	15 (38.5%)	0.0005	5.35 (2.14–12.98) *
		Dose (mg/kg)	0.2 (0.2–0.4)	0.2 (0.2–0.4)	0.24	
	12–24 hours	At least one dose administrated (n. of dogs)	6/86 (7%)	6 (15.4%)	0.19	
		Dose (mg/kg)	0.3 (0.2–0.4)	0.2 (0.2–0.4)	> 0.99	

*n.* number; *min* minute; *NSAID* non-steroidal anti-inflammatory drug

**Table 3:** Intra- and postoperative complications of 132 dogs undergoing thoracolumbar (cranial to the 1st lumbar vertebra) or lumbar (caudal to the 1st lumbar vertebra) and in which a bilateral erector spinae block (group ESPB) or a fentanyl infusion (group CRI) was used. Odd ratio (95% CI) is reported when appropriate

Group			ESPB (n = 93)	CRI (n = 39)	ESPB vs CRI ***p*** value	OR (95% CI)
Information						
Intraoperative complications	Hypotension (n.)		34 (36.6%)	16 (41%)	0.69	
	Hypothermia (n.)		74 (79.6%)	24 (61.5%)	0.04	2.57 (1.13–5.74)
	Others	Regurgitation (n.)	4 (4.3%)	0 (0%)	0.34	
		2nd degree AV block (n.)	3 (3.2%)	2 (5.1%)		
		Vagal reflex (n.)	1 (1.1%)	0 (0%)		
		Haemorrhage < 5% (n.)	1 (1.1%)	0 (0%)		
		Bradycardia (n.)	1 (1.1%)	0 (0%)		
Postoperative complications	MDR bacterial infection (n.)		1 (1.1%)	0 (0%)	1	

*n.* number of dogs; *AV* atrio-ventricular; *MDR* multi-drug resistant

The minimum alveolar concentration (MAC) multiple of isoflurane (IsoFlo; Zoetis UK Ltd., UK) or sevoflurane (SevoTek; Animalcare, UK) delivered to maintain anaesthesia was calculated by averaging the percentage of the end-expiratory fraction of the inhalational anaesthetic agent (FE′Iso or FE′Sevo, respectively) recorded every 5 min (Mindray BeneView T5; Mindray, UK). The average was further divided by the MAC of isoflurane and sevoflurane in dogs: 1.28 and 2.36%, respectively [16, 17].

Intraoperative hypotension was defined as MAP < 60 mmHg for two consecutive readings, taken at five-minute intervals, using an oscillometric technique [18]. Bradycardia was defined as HR < 50 beats *per* minute with concurrent hypotension treated administering an antimuscarinic. Hypothermia was defined as oesophageal temperature < 36.5 °C for 10 consecutive minutes [19]. Any other anaesthetic and surgical intraoperative complication was also recorded. The anaesthetist in charge of each case was aware if a bilateral ESPB was performed or not; hemilaminectomies were performed by more than one neurosurgeon.

Postoperative pain was evaluated every 2 h using the short form of the Glasgow Composite Measure Pain Scale (CMPS-SF) and methadone (0.2 mg/kg IV; Comfortan; Dechra Pharmaceuticals, UK) was administered if pain score was ≥5 out of 20 [20]. Pain scores were performed by dedicated neurology nurses trained and familiar with the CMPS-SF. They were aware if an ESBP was performed or not. Methadone requirement during the first 6, 6–12 and 12–24 postoperative hours was compared between groups. Postoperative complications and other analgesic drugs administered were also recorded.

### Statistical analysis

Considering that group F consisted of 39 dogs, at least 79 dogs were needed in group ESPB to test our main hypothesis with an effect size of 0.5, an alpha error of 0.05 and beta of 0.08, and considering an allocation ratio of 0.5 between groups (G*power 3.1 for MAC).

D’Agostino & Pearson test was used to assess data distribution (GraphPad Prism version 8 for Mac, GraphPad Software Inc., CA, USA). Depending on their distribution, continuous data were analysed with either Student’s t test or Mann-Whitney U test and reported as mean ± standard deviation or median (range), respectively.

Fisher’s exact test was used to compare categorical data. Odds ratio (OR) or reciprocal OR with 95% confidence interval (CI) were calculated and reported when appropriate. A value of *p* < 0.05 was considered statistically significant.

## Results

**Fig. 1:**
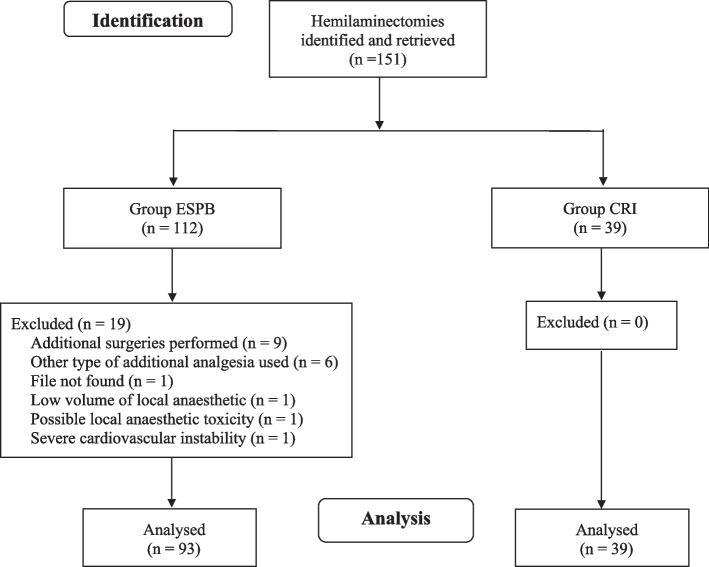
The Consolidated Standards of Reporting Trials (CONSORT) flow diagram. Abbreviations: n, number

Overall, 112 hemilaminectomies in which an ESPB was performed were found between June 2019 and August 2020: 19 cases were excluded, and 93 cases were compared to the 39 cases of CRI group (Fig. 1). No missing data for each variable of interest was found in the included cases. In group ESPB, 10 dogs (7.6%) were excluded from the postoperative analysis because their behaviour made pain assessment impossible, and methadone was administered every 4 h. One dog was not included in the study as sinus arrest occurred 10 minutes after a thoracic (T13) ESPB performed administering 1 mL/kg of 0.125% levobupivacaine to each side (2.4 mg/kg in total). As no improvement was noticed after multiple atropine administrations (0.02 mg kg^− 1^ IV; Atropine Sulfate, Martindale Pharma, UK), an intralipid solution (1.5 mL/kg followed by 0.25 mL/kg/minute; Intralipid 20%, Fresenius Kabi Ltd., UK) was administrated IV and sinus rhythm was re-established [21].

No difference in demographics were found between groups, but dogs in group ESPB were 5.87 times more likely to have non-ambulatory paraparesis at presentation (MFS = 3; Table 1). The pre-anaesthetic medications, induction agents and inhalational anaesthetic agents administered are reported in Table 1. Dexmedetomidine was administrated to 95.7% of the dogs in group ESPB compared to none in group CRI (*p* < 0.0001). The MAC multiple of inhalational anaesthetic agent administered was lower in group ESPB (*p* < 0.0001; Table 1).

Preoperative magnetic resonance imaging (MRI) was performed in 87.9% of dogs; in the remaining 12.1% of dogs (group ESPB) MRI was performed the day before surgery. Disc extrusion was diagnosed in 98.5% of dogs with no difference in lateralisation between groups (Table 1). In 90.1 and 100% of dogs a single disc extrusion was diagnosed in group ESPB and CRI, respectively (*p* = 0.01). No difference between groups was found when comparing the area of surgery, but a higher number of discs were fenestrated in group CRI (*p* < 0.0001; Table 1). Only one neurosurgeon operated on 70.4% of the dogs included in this study: 100% of dogs in group CRI and on 58% of dogs in group ESPB. The remaining dogs were operated on by other two neurosurgeons previously trained by at our institution. While surgical time was similar between groups, anaesthesia time was longer (*p* < 0.01) in group ESPB (Table 1).

The bilateral ESPB was performed by several anaesthetists using 0.125% (0.12–0.25) levobupivacaine. The volume of levobupivacaine administered to each side was 1 (0.5–1.7) mL/kg while the total dosage used was 2.3 (1.2–2.5) mg/kg. Overall, the number of dogs in which at least one rescue fentanyl bolus was administered during surgery was not different between groups. However, in group CRI, dogs were 4.25 times more likely to receive a rescue fentanyl bolus during lumbar hemilaminectomy (Table 2). The median number (*p* = 0.006), but not the overall amount (μg/kg/hour; *p* = 0.59), of rescue fentanyl boluses administered was higher in group CRI (Table 2). The time from start of surgery to the first rescue fentanyl bolus administration was shorter in group CRI (*p* < 0.0001; Table 2). During phase 1 of surgery, rescue fentanyl boluses were administered in 23.7% of dogs (22 out of 93) in group ESPB and in 53.9% of dogs (21 out of 39) in group CRI [*p* = 0.001; reciprocal OR (95% CI) = 3.77 (1.69–8.02)]. During phase 2 of surgery, rescue fentanyl boluses were administered in 31.2% dogs (29 out of 93) in group ESPB and in 2.6% dogs (1 out of 38) in group CRI [*p* = 0.0002; OR (95% CI) = 17.22 (2.93–181.3)].

In group ESPB, no differences in the total levobupivacaine volume [1.9 (1–2.4) mL/kg and 1.9 (1–2.1) mL/kg; *p* = 0.85) and concentration [0.125% (0.12–0.25) and 0.125% (0.12–0.25); *p* = 0.31] were found between dogs in which a rescue fentanyl bolus was and was not administered, respectively. Comparing the area of surgery within group ESBP, rescue fentanyl boluses were administered in 59.7% of dogs (37 out of 62) during thoracolumbar and in 45.2% of dogs (14 out of 31) during lumbar surgery [*p* = 0.19; reciprocal OR 1.80 (0.75–4.06)].

Prior to wound closure, morphine (0.1 mg/kg; Morphine Sulphate; Martindale Pharma, UK) or buprenorphine (0.01 mg/kg; Buprevet, Virbac, France), with or without methylprednisolone (0.4 mg/kg; Depo-Medrone, Pfizer, Belgium), was splashed extradurally to 83 dogs (91.2%) in group ESPB, but to none in group CRI.

Postoperatively, a non-steroidal anti-inflammatory drug (NSAID) and gabapentin (10 mg/kg per os three time a day; Gabapentin, Milpharm, UK) was administered to 100% of dogs in group CRI and 76.3% of dogs in group ESPB (*p* = 0.0008; Table 2). Of the remaining dogs in group ESPB, a different combination of oral analgesic was used (Table 2). A dexmedetomidine (0.5–1.5 μg/kg/hour; Dexdomitor, Orion Pharma, UK) or lidocaine (30 μg/kg/minute; Lidocaine hydrochloride, Halmen Pharmaceutical, UK) infusion was administered postoperatively to 4 and 2 dogs in group ESPB, respectively. In 20 out of 39 dogs in group CRI, two 1.5 cm wide 5% lidocaine patch (Versatis 5%; Grünenthal, Germany) strips were cut to the same length as the incision and applied to the skin along both sides of the surgical wound [15].

The CMPS-SF was used to guide postoperative methadone administration in 92.4% of dogs (122 out of 132). No postoperative methadone was administered to 73.1% of dogs in group ESPB. During the first 6 and 6–12 postoperative hours, the number of dogs that required at least one dose of methadone was higher in group CRI (*p* = 0.0035 and *p* = 0.0005, respectively). No difference in postoperative methadone requirement was found during the 12–24 postoperative hours (Table 2).

Intra- and postoperative complications are reported in Table 3. In group ESPB, dogs were 2.57 times more likely to become hypothermic (*p* = 0.04).

## Discussion

This study retrospectively compared the analgesic efficacy of a bilateral ESPB versus a fentanyl CRI in dogs undergoing hemilaminectomy. While the overall number of dogs receiving at least one rescue fentanyl bolus was similar between groups, the number of rescue fentanyl boluses administered was higher in group CRI, especially during lumbar hemilaminectomy. A rescue fentanyl bolus was more likely to be administered during phase 1 of surgery in group CRI, and phase 2 of surgery in group ESPB. Postoperatively, a significant methadone sparing effect was found during the first 12 hours in group ESPB. These findings support our initial hypothesis and agree with a previous study in which a unilateral ESPB block reduced the perioperative opioid consumption and intraoperative adjuvant analgesic administration in dogs undergoing hemilaminectomy [13]. However, interpretating our results, we need to acknowledge several limitations. The retrospective nature of the study, and the use of a control group obtained from a previous prospective study performed at our institution few years before, might have affected the standardisation of the anaesthetic and analgesic management between groups. Despite all the neurosurgeons used a similar surgical technique, a different tissue handling caused by a different level of experience gained over these years could have affected the degree of surgical trauma, the level of nociception and the postoperative pain. Multiple anaesthetists with different level of experience in ultrasound guided regional anaesthesia were involved, and the volumes and concentrations of local anaesthetic administered were at their discretion. This variability might have affected the effectiveness of the ESPB and our results, despite junior anaesthetists were always supervised by more senior one during the ESPB execution. Rescue fentanyl boluses were administered by the attending anaesthetist according to his/her clinical judgement. Despite the reason for fentanyl administration was reported in the anaesthetic record, we cannot categorically exclude that some nociceptive events were missed or misinterpreted, especially considering the different level of experience of each anaesthetist involved in the study. A different level of preoperative pain between groups could have affected the postoperative results [22], but this is in our opinion unlikely especially considering that dogs in group ESPB had a higher MFS, therefore a more severe spinal cord compression. Several anaesthetists and ward nurses, with different level of experience and aware if an ESPB was performed, assessed postoperative pain. This, together with the not standardised postoperative management, could have added an element of bias affecting our results. However, the CMPS-SF, that was used during the entire study period at our institution, was clinically validated between three different institutions to produce a score linked to the requirement of additional analgesia, rather than to measure the actual level of pain [20]. Despite all these limitations, we believe that the results of this study might have a clinically value, as they represent a real clinical scenario of single referral centre.

Dogs in group ESPB were less likely to receive rescue fentanyl boluses during phase 1 of surgery. This result could be explained considering that in canine cadaveric studies the injection of dye into the thoracic and lumbar ESPB interfascial plane resulted in staining of the dorsal branches of the spinal nerves [3, 5, 7, 8]. These branches innervate the vertebral laminae, articular facets, epaxial muscles and skin near the dorsal midline [23, 24]. During phase 2 of surgery, when the spinal cord was manipulated and the herniated disc removed, or adjacent discs fenestrated, less dogs required rescue fentanyl boluses in group CRI. The structures within the vertebral canal are innervated by the meningeal branch of the spinal nerves in humans and although, the canine thoracolumbar region lacks a meningeal ramus per se, they are innervated [23, 25]. Therefore, to be effective on the meningeal branches, the local anaesthetic injected in the erector spinae fascial plane should spread paravertebrally and extradurally, as demonstrated by some human cadaveric studies, but neither stain has been found in dogs when a parasagittal approach was used [1, 3, 5, 7, 8]. Previously, several dogs required analgesic interventions when the proximity of the dorsal root ganglion was manipulated [13]. Therefore, unsurprisingly, fentanyl CRI was more effective than the ESPB during phase 2 of surgery. Unexpectedly, 64 out of 93 dogs (68.8%) in group ESPB did not require rescue fentanyl administration during disc removal and fenestrations, even if the number of dogs with more than one disc extrusion was higher. Probably, the ESPB mechanism of action is more sophisticated than what has been postulated in dogs so far [3, 5, 8], and differences between cadaveric studies and the clinical performance of the block should be considered.

In comparison to Portela and colleagues (2021), a bilateral rather than unilateral ESPB was performed, as the cutaneous and vertebral canal nerves can cross or communicate across the midline [13, 23, 26]. Furthermore, a different volume and concentration of local anaesthetic was used in the present study. Theoretically, when performing an interfascial plane block, the greater the local anaesthetic volume and concentration, the higher the success rate [2, 27]. However, no direct correlation between the volume, concentration and intraoperative efficacy has been clinically established in humans [27, 28]. In our study, a high volume and low concentration were employed (1 mL/kg of 0.125% local anaesthetic *per* site). On the contrary, a low volume and high concentration [median (range) of 0.46 (0.2–0.6) mL/kg of 0.5% bupivacaine] was previously used [13]. Our choice was made considering that the anulus fibrosus of a disc might receive innervation from the spinal nerves as far as two segments cranially and caudally [23]. Furthermore, we considered the length of the skin incision and the generally high number of fenestrations performed at our institution, which were greater than those performed in Portela and colleagues’ study (2021) [13]. Despite these technical differences, the overall prevalence of rescue analgesia administration between the two studies (54.8 and 59.5%) was similar. A prospective randomised clinical trial using different local anaesthetic volumes and concentrations, and comparing unilateral with bilateral, is warranted to better understand the intraoperative effect of an ESPB in dogs undergoing hemilaminectomy.

The MAC multiple of inhalational anaesthetic agent administered was lower in group ESPB. The real clinical value of this result is debatable, if we consider that the FE′Iso was maintained constant in group CRI, and the anaesthetist was probably biased by the knowledge of a performed ESPB. Furthermore, dexmedetomidine was used as pre-anaesthetic medication in 95.7% of dogs in group ESPB. When medetomidine (5 μg/kg IV) was administrated to isoflurane anaesthetised dogs, MAC multiples were similar to baseline values by 60 minutes from administration [29]. In addition, Muir and colleagues (1999) observed a lack of analgesic effect with intramuscular medetomidine at doses of 2–5 μg/kg in dogs [30]. Furthermore, the analgesic effects of intravenous dexmedetomidine were noted at doses above 2 μg/kg in dogs [31]. Considering that the median dose of dexmedetomidine used was 1 μg/kg, and the median time from premedication to start of surgery was 85 minutes, we believe that it is unlikely that administration of dexmedetomidine contributed to decrease the MAC multiple of inhalational anaesthetic agents and to lower the number of rescue fentanyl boluses administered to group ESPB.

Overall, the postoperative methadone requirement was reduced in group ESPB, as reported by previous human and veterinary medicine studies [13, 32, 33]. During the first 12 hours methadone requirement was reduced, but no difference was found between 12 and 24 hours. Therefore, it is plausible that the effect recorded was related to the duration of the levobupivacaine [34]. Nevertheless, the postoperative analgesic protocol was not standardised, and multiple contributing factors should be considered. In group ESPB, for example, 91.2% of the dogs had an opioid, with or without a steroid, splashed extradurally before the end of surgery, and this could have affected the postoperative methadone requirement. In fact, while extradural opioid splash provided additional postoperative analgesia in dogs after hemilaminectomy [35], extradural steroids might have decreased the possible spinal cord inflammation associated with a herniated disc [36]. All dogs in group CRI received a NSAID, while it was only administered to 78.5% of dogs in group ESPB. A lidocaine patch was applied to 51% of the dogs in the group CRI, but this should not have impacted our results as this technique did not provide any extra postoperative analgesia following hemilaminectomies in dogs [15].

Different from our results, a unilateral ESP block produced postoperative benefits up to 48 hours in dogs after hemilaminectomy [13]. This might be related to the greater local anaesthetic concentration administered; in humans, greater reduction in postoperative rescue analgesia was found when higher concentration of local anaesthetic was used for the ESPB [28]. However, while a non-validated pain scoring system was previously used [13], the pain scale employed in our study has been validated to assess acute postoperative pain in dogs [20]. In addition, dexmedetomidine, used as an adjuvant to the local anaesthetic in 21% of their dogs [13], might have prolonged the ESPB effect [37], and the intraoperative infusion of lidocaine and/or ketamine to 23.8% might also have affected Portela’s and colleagues’ results.

General anaesthesia time, but not surgical time, was longer in group ESPB. Unfortunately, the time to execute the ESPB was not measured, but it might have prolonged the overall anaesthetic time. A higher prevalence of hypothermia was found in group ESPB. While this might be the result of a prolonged anaesthetic time, no significant association was previously found between anaesthetic time and hypothermia in dogs undergoing hemilaminectomies [38]. It could also be possible that the bilateral ESPB caused a pronounced peripheral vasodilation, promoting heat loss and hypothermia [39].

A dog developed sinus arrest 10 minutes after a T13 ESPB and it was not responsive to antimuscarinic therapy and intralipid solution was administered as local anaesthetic systemic toxicity (LAST) was suspected [21]. However, we cannot categorically exclude that the response to the intralipid solution was coincidental. In humans, LAST was associated with an ESPB in 1.6% of cases but was described only as a central nervous system toxicity [27, 40]. It might develop following a local anaesthetic spread to the paravertebral and intercostal space, and a fast systemic absorption due to the high vascularisation of these areas [27]. However, while no correlation between LAST and the local anaesthetic volume was noted, LAST occurred mainly by administering low local anaesthetic concentrations [27], as used in this study. No evidence of LAST was found in a previous study using higher concentrations of local anaesthetic [13].

## Conclusions

Under the conditions of this study, and compared to fentanyl CRI, a preoperative bilateral ultrasound-guided ESPB in dogs undergoing hemilaminectomy appears to be useful to limit the number of dogs requiring rescue fentanyl boluses administration, particularly from the skin incision to the end of vertebral lamina drilling. Postoperatively, a bilateral ESPB may reduce the opioid consumption during the first 12 hours. However, the different postoperative management strategies impair our ability to draw any firm conclusion regarding the postoperative effect of the ESPB. Prospective studies are warranted to validate our observations.

## Data Availability

All data generated and/or analysed during this study are included in this published article.
